# Diagnosing Hunter syndrome in pediatric practice: practical considerations and common pitfalls

**DOI:** 10.1007/s00431-012-1703-y

**Published:** 2012-03-01

**Authors:** Barbara K. Burton, Roberto Giugliani

**Affiliations:** 1 Division of Genetics, Birth Defects and Metabolism, Children’s Memorial Hospital, 2300 Children’s Plaza, Chicago, IL 60614 USA; 2 Medical Genetics Service, HCPA, Department of Genetics, UFRGS, Porto Alegre, Rio Grande do Sul Brazil; 3 INAGEMP, Porto Alegre, Rio Grande do Sul Brazil

**Keywords:** Lysosomal storage disease, Mucopolysaccharidosis II, Hunter syndrome, Glycosaminoglycans, Iduronate-2-sulfatase, Diagnosis

## Abstract

Mucopolysaccharidosis II (MPS II), or Hunter syndrome, is an X-linked lysosomal storage disorder caused by a deficiency in the enzyme iduronate-2-sulfatase. Affected patients suffer progressive damage to multiple organ systems and early mortality. Two thirds of patients also manifest cognitive impairment and developmental delays. MPS II can be extremely difficult to diagnose before irreversible organ and tissue damage has occurred because of an insidious onset and the overlap in signs and symptoms with common childhood complaints. This is particularly true of patients without cognitive impairment (attenuated phenotype). Although not curative, early treatment with enzyme replacement therapy before irreversible organ damage has occurred may result in the greatest clinical benefit. Here, the signs, symptoms, and surgical history that should trigger suspicion of MPS II are described, and the diagnostic process is reviewed with a focus on practical considerations and the avoidance of common diagnostic pitfalls. Once a diagnosis is made, multidisciplinary management with an extended team of pediatric specialists is essential and should involve the pediatrician or family practice physician as facilitator and medical home for the patient and family. *Conclusion*: Because routine newborn screening is not yet available for MPS II, the involvement and awareness of pediatricians, family practice physicians, and pediatric specialists is critical for early identification, diagnosis, and referral in order to help optimize patient outcomes.

## Introduction

The mucopolysaccharidoses (MPS) are a group of rare genetic disorders within the larger family of lysosomal storage diseases (LSDs). Each MPS disorder is caused by a deficiency in the activity of a specific lysosomal enzyme required for the degradation of glycosaminoglycans (GAGs). The resulting accumulation of GAGs within lysosomes produces progressive cellular damage, leading to organ failure and reduced life expectancy [32].

Mucopolysaccharidosis II (MPS II or Hunter syndrome; OMIM 309900) is an X-linked disorder with an incidence of 0.3 to 0.71 per 100,000 live births [24]. It is a caused by a deficiency in the lysosomal enzyme iduronate-2-sulfatase (I2S), leading to an accumulation of the GAGs dermatan sulfate and heparan sulfate [2]. The consequence of GAG accumulation is progressive, multi-organ disease (Tables 1 and 2).

**Table 1:** “Red Flag” signs and symptoms of MPS II that occur early in the disease course

Coarse facial features (may be subtle in the attenuated phenotype)
Recurrent respiratory infections
Chronic rhinorrhea
Upper airway restriction/noisy breathing/snoring
Recurrent otitis media
Hearing loss
Heart murmur
Hepatomegaly
Umbilical and inguinal hernia
Recurrent watery diarrhea
Joint stiffness
Developmental delay and/or speech delay (in severe phenotype *only*)

**Table 2:** Signs and symptoms of MPS II and clinically similar LSDs

Sign/symptom	MPS II	MPS I	MPS III	MPS VI	MPS VII	Sialidosis type 2	Mucolipidosis II alpha/beta	Mucolipidosis III alpha/beta	Alpha-mannosidosis
Coarse facial features	++	++	+	++	++	++	++	+	++
Macrocephaly	++	++	+	++	++	++	−	−	++
Communicating hydrocephalus	+	+	−	+	+	−	+	++	++
Dental abnormalities	++	+	−	++	++	−	++	−	++
Cognitive/developmental delay	+	+	++	−	++	++	++	+	++
	Severe patients only	Severe patients only							
Spinal cord compression	+	+	−	+	+	−	+	++	−
Carpal tunnel syndrome	++	++	−	++	++	−	+	++	−
Hyperactivity, aggression, impulsivity	+	−	++	−	−	−	−	−	−
Seizures	+	−	+	−	−	++	−	−	−
Hearing loss	++	++	+	++	++	++	++	++	++
Recurrent ear infections	++	++	+	++	++	−	++	++	++
Persistent rhinorrhea	++	++	+	++	++	−	−	−	−
Frequent respiratory infections	++	++	+	++	++	−	++	++	++
Respiratory obstruction	++	++	−	++	++	−	++	−	−
Sleep apnea	++	++	+	++	++	−	++	−	−
Hepatosplenomegaly	++	++	+	++	++	++	++	++	++
Umbilical hernia/inguinal hernias	++	++	+	++	++	++	++	++	−
Chronic diarrhea	++	+	+	−	−	−	−	−	−
Dysostosis multiplex	++	++	+	++	++	++	++	++	++
Growth retardation	++	++	−	++	++	++	++	++	++
Claw hands	++	++	−	++	++	−	++	++	+
Joint stiffness/contractures	++	++	+	++	++	−	++	++	++
Cardiac valve disease	++	++	+	++	++	−	++	++	−
Corneal clouding	−	++	−	++	++	++	++	++	−

“*++*” Exhibited by majority of patients with diagnosis, “+” exhibited by some patients with diagnosis, “*−*” not exhibited

MPS II is characterized by clinical heterogeneity in that the number and type of presenting signs and symptoms can vary widely among patients. It is often described as having two phenotypes: attenuated and severe. Patients with either phenotype generally appear normal at birth. In patients with the severe phenotype, clinical signs and symptoms (Tables 1 and 2) usually emerge between 2 and 4 years of age [47], whereas in those with the attenuated phenotype, signs and symptoms may not emerge until late childhood or early adolescence [48]. Patients with attenuated and severe disease both experience significant somatic signs and symptoms, but patients with severe disease also have profound cognitive impairment and developmental regression [49, 50] which is not seen in the attenuated phenotype. Life expectancy is also shorter in patients with severe disease, with death typically occurring in the second decade of life [15]. Patients with the attenuated phenotype may survive into adulthood, although premature mortality does occur [15]. It is important to note that although patients with attenuated disease do not experience cognitive impairment, they may still demonstrate all of the somatic signs and symptoms of the disease, including neurological complications such as communicating hydrocephalus, spinal cord compression, and hearing loss [24].

Historically, treatment for MPS II has been supportive; however, enzyme replacement therapy (ERT) with idursulfase, a recombinant human I2S enzyme (Elaprase®, Shire Human Genetic Therapies, Inc., Cambridge, MA), is now available in more than 40 countries. In a phase II/III clinical trial enrolling children over the age of 5 years, idursulfase was shown to improve some of the somatic signs and symptoms of the disease, including walking ability, although it does not affect the cognitive decline seen in patients with severe disease [29]. The most common adverse events are infusion reactions, such as fever, flushing, rash, or headache. Life-threatening anaphylactic reactions have occurred in patients receiving ERT. A clinical trial of idursulfase for the treatment of MPS II patients aged 5 years and under is currently ongoing.

Early initiation of ERT may offer the greatest benefit [28, 38], but there is often a delay of several years between the onset of signs and symptoms and diagnosis. This is particularly true for patients with the attenuated phenotype, as the disease onset can be insidious (see Case study: patient D). Such delays increase the risk of irreversible organ damage and may decrease the benefit of ERT [28, 43, 47]. Because it is the pediatrician or family practice physician who often recognizes the existence of a deeper problem, increased awareness of MPS II is a critical factor in early diagnosis, referral, and treatment. The recently published European recommendations for the management of MPS II note that expertise in diagnosis and managing MPS II varies widely throughout Europe [37]. The authors highlight the need for guidance on how to recognize and diagnose this disorder. A previous review offers an excellent overview of the disease for the reader who is unfamiliar with MPS II [24]. Here, we focus on the practical considerations of diagnosing this syndrome in the busy pediatric practice by describing “red flags” in the patient history, suspicious signs and symptoms, and common diagnostic pitfalls to avoid. We discuss the available laboratory tests and whether or not they can be relied upon to confirm or rule out the diagnosis, and outline the differential diagnoses. We conclude with a diagnostic algorithm specifically tailored for the pediatric general practice.

## Clinical suspicion of MPS II

**Fig. 1:**
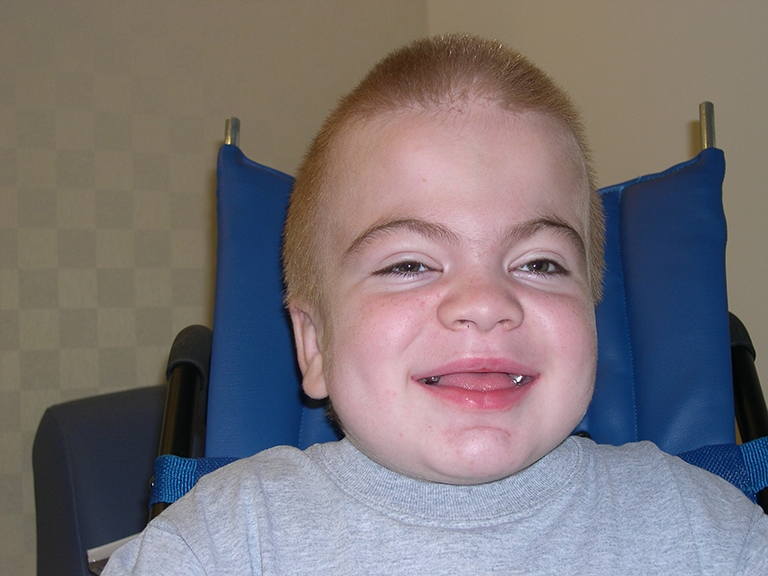
Coarse facies in a 5-year-old child with MPS II. Note the frontal bossing, heavy eyebrows, puffy eyes, broad “saddle” nose, large jowls, thick lips, and enlarged tongue

**Fig. 2:**
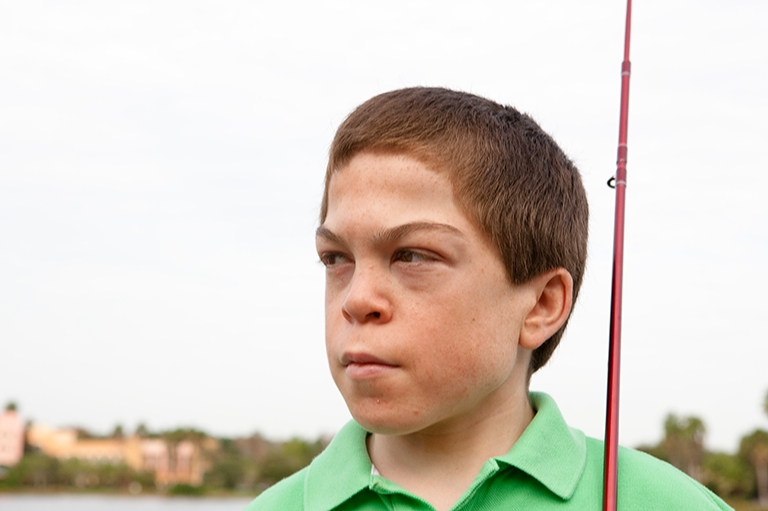
Patient D (attenuated phenotype) at 13 years of age. Note that the coarse facies are more subtle than seen in patients with the severe phenotype

**Fig. 3:**
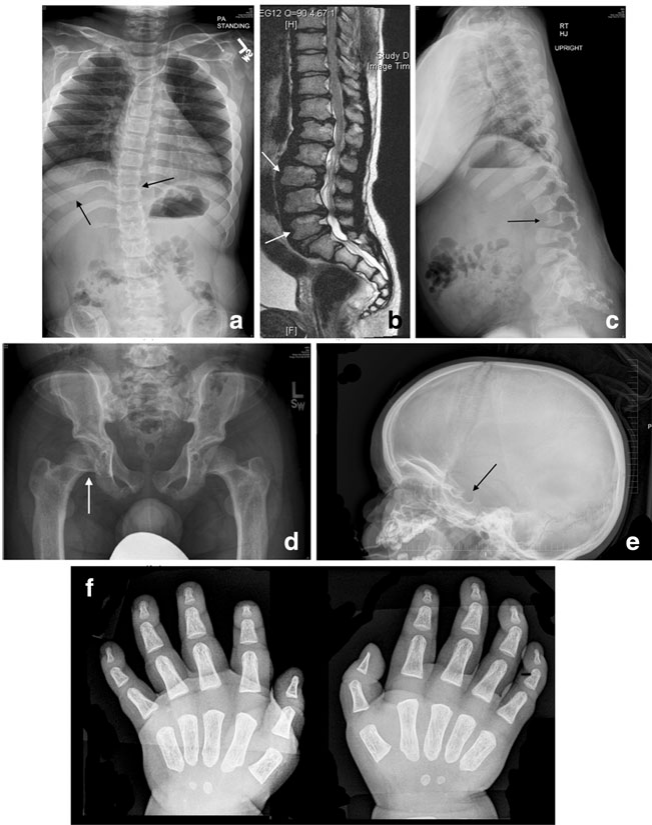
Skeletal manifestations of MPS II: **a** 33° scoliosis with coarseness of the bones and widening of the ribs in a 16-year-old patient with attenuated MPS II; **b** magnetic resonance imaging demonstrating mild thoracolumbar kyphosis and typical anterior vertebral body deficiency in a 16-year-old patient with attenuated MPS II; **c** significant thoracolumbar kyphosis in a 4-year-old patient with severe MPS II; **d** caput valgum and mild femoral head uncoverage in a 9-year-old patient with severe MPS II; **e** J-shaped sella in a 21-month-old patient with MPS II; **f** severe delay in maturation compatible with 3–6 months of age, mild proximal tapering of the metacarpals, and minimal slenderness of the distal phalanges in a 21-month-old patient with MPS II. Images **a**–**d** reproduced with permission from White et al. [45]

Early recognition of MPS II requires careful attention to the presence of multiple signs and symptoms, many of which overlap with common childhood complaints. Clinical suspicion of the disease can be triggered by particular clusters of signs and symptoms that are unlikely to appear in an unaffected child but that often occur together in the child with MPS II (Table 1). Most of the common somatic manifestations that occur early in the disease course appear in both the attenuated and severe phenotypes, although they may be more subtle in the attenuated phenotype [24]. Coarse facial features are a strong diagnostic clue of an LSD and are manifest in MPS II patients with varying disease severity (Figs. 1 and 2). Ear, nose, and throat signs and symptoms include recurrent respiratory infections, chronic rhinorrhea, upper airway obstruction, noisy breathing and snoring, hearing loss, and recurrent otitis media [16, 31]. Some children with MPS II will have a detectable heart murmur relatively early in the disease course [7]. Common abdominal complaints include hepatomegaly, umbilical and inguinal hernias without family history of hernia, and recurrent episodes of diarrhea [24]. These episodes of watery diarrhea are frequently observed in the MPS II population, although the underlying pathophysiology is not understood. The diarrhea is often very difficult for the family to manage and is resistant to medication. Early skeletal signs include joint stiffness and restricted range of motion [19]. Evidence of dysostosis multiplex can usually be seen on X-ray (Fig. 3). Although by later in childhood patients with MPS II have growth restriction and short stature, it is important to point out that in early childhood, children with MPS II often grow faster than normal. Height and weight may be near or over the top of the standard growth curves. The slowing in growth that is associated with the disease only begins at around 3 to 4 years of age [36].

The presence of several of these somatic signs or symptoms should prompt suspicion of MPS II. Keep in mind that most patients do not display all of them early in the disease course, and manifestations like coarse facial features may be very subtle (Fig. 2). In addition to somatic signs and symptoms, patients with the severe phenotype will begin to display developmental delay and/or speech delay between the ages of 2 to 5 years [47] and will eventually progress to severe neurological and cognitive decline by the second decade of life [49, 50].

A history of frequent and recurrent surgeries is a defining characteristic of MPS II and may also trigger clinical suspicion of the disease. A study that analyzed surgical data from 527 patients with MPS II found that surgical interventions were performed in 83.7%, with a median number of 3.0 operations per patient [26]. The median age at first operation was 2.6 years, and most patients (57%) underwent at least one surgical procedure before diagnosis. Tympanostomies, repairs of inguinal hernias, and operations for carpal tunnel syndrome were performed in a greater proportion of the study population than the general population. Thus, repeated early surgeries of these types should serve as a clinical “red flag,” prompting careful examination for other features of MPS and appropriate diagnostic testing.

### Avoiding pitfalls

A common pitfall when examining the patient with undiagnosed MPS II is a failure to link the many, seemingly unrelated signs and symptoms experienced by the patient into a single syndrome. This may be particularly challenging in cases of attenuated MPS II in which the onset of disease signs and symptoms is insidious, facial dysmorphisms may be mild, and no developmental delay is seen (see Case study: patient D).

A second common pitfall in recognizing MPS II is relying too heavily upon exact diagnoses of LSDs in the family history. In the past, LSDs were often diagnosed clinically. It was not uncommon for MPS disorders to be lumped together as “Hurler syndrome”, which we now know to be the severe phenotype of MPS I. A family history of “Hurler syndrome” or other MPS disorder should not rule out testing for MPS II if warranted by findings on examination. The authors are aware of several cases where a family history of “Hurler syndrome” in an uncle or other male relative has delayed the diagnosis of MPS II in an affected child.

## Case study: patient D

Patient D, a Caucasian male, was born at full term with no abnormal signs and symptoms. At 10 weeks of age, the pediatrician diagnosed bilateral inguinal hernias which were repaired surgically. Throughout the patient's first 5 years of life, he presented with chronic watery diarrhea, frequent upper respiratory infections, and repeated bouts of acute otitis media that required antibiotic treatment. No developmental or motor delays were noted. The parents would, on occasion, express their concerns that something was “wrong” with the child, but each time the parents were reassured that everything was fine. By 5 years of age, patient D was beginning to show some very mild facial dysmorphisms, and his growth began to slow. He also showed some lack of flexibility in the upper body. The parents again raised their concerns that something was amiss, but again the pediatrician reassured them. Over the next 3 years, patient D's growth slowed considerably and his range of motion was further limited. He was also diagnosed with moderate hearing loss and received a hearing aid. Because of his range-of-motion deficits and slow growth, the pediatrician referred the patient to a pediatric orthopedist. The orthopedist noted the child's stiff joints, slow growth, short stature, mild facial dysmorphisms, hearing loss, and history of frequent respiratory infections, and he suspected an MPS disorder. The orthopedist referred the patient to a clinical geneticist, and laboratory tests confirmed the diagnosis as MPS II. The child was 8 years old at the time of diagnosis (Fig. 2), despite the fact that signs and symptoms of MPS II were present as early as the first months of life.

### MPS II in females

A diagnosis of MPS II cannot be ruled out based on female sex. Unlike the other X-linked LSDs (Fabry disease, Danon disease), females carrying one mutated allele of the gene that codes for I2S (*IDS*) are, indeed, usually asymptomatic. However, because MPS II has been documented in a few females, MPS II should not be ruled out based on sex or inheritance patterns alone. Most commonly, symptomatic MPS II in females occurs through skewed X-chromosome inactivation, in which the mutated allele of *IDS* is preferentially activated and the normal allele is inactivated [41]. It can also be observed in females with a 45,X karyotype or with an X-chromosome rearrangement, so chromosome analysis should always be performed in a female with a confirmed diagnosis of MPS II. Females with this disorder typically present with a severe phenotype [41]. Biochemical and genetic explanations have been reviewed by Pinto et al. [34].

### Differential diagnosis

Key “red flag” signs and symptoms of MPS II are presented in Table 1. However, because MPS II can present on a spectrum of severity with variable signs and symptoms, the differential diagnosis based on clinical features can be very challenging. For example, several LSDs share features with MPS II (Table 2); these include MPS I (Hurler syndrome) [30], MPS VI (Maroteaux–Lamy syndrome) [42], MPS VII (Sly syndrome) [32], sialidosis type 2 [14], mucolipidosis II alpha/beta [3], mucolipidosis III alpha/beta [3], and α-mannosidosis [22]. The LSDs fucosidosis [46] and multiple sulfatase deficiency [11] also share signs and symptoms with MPS II, although these two disorders are extremely rare. Because so many clinical features are shared among these LSDs, laboratory testing is necessary when clinical suspicion of an MPS disorder has been raised.

## Laboratory diagnostic testing for MPS II

**Fig. 4:**
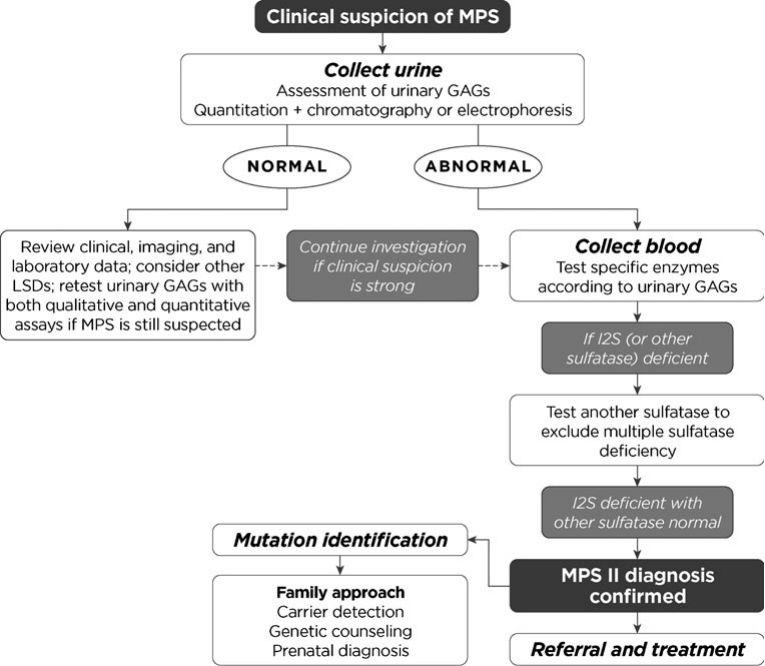
Diagnostic algorithm for MPS II

A common diagnostic algorithm for MPS II is presented in Fig. 4. Importantly, patients with MPS-like signs and symptoms should be promptly referred to a clinical geneticist even if the standard diagnostic assays for MPS indicate normal results. Further testing may be indicated to rule out another LSD (Table 2).

### Urinary GAG excretion

The level of urinary GAGs is increased in patients with any MPS, so the detection of excessive urinary GAG excretion is generally the first diagnostic approach, although patients with a family history of MPS II should proceed directly to enzyme activity assays and/or molecular genetic analyses [32]. Assays can be divided into semiquantitative (the Berry spot and Ames spot tests) and quantitative (the carbazole reaction, cetylpyridinium turbidity test, alcian blue reaction, dimethylmethylene blue test, and azure A and B method). Although simple and inexpensive, semiquantitative assays are not reliable due to a relatively high rate of false positives and false negatives [8, 20] Improper reliance on semiquantitative assays alone thus represents a major pitfall in the diagnostic process for the MPS disorders. Among the quantitative assays, the carbazole reaction has a low sensitivity for MPS IV (Morquio syndrome) and the cetylpyridinium turbidity test has a low sensitivity for MPS IV and MPS III (Sanfilippo syndrome); therefore, these assays should be avoided [5, 35].

All samples should also be analyzed via thin layer chromatography or one-dimensional or bidimensional electrophoresis for the identification of an abnormal GAG pattern even if the overall uGAG level is not elevated [24]. The presence of excess dermatan sulfate and heparan sulfate in urine is indicative of MPS I, MPS II, or MPS VII. Excess heparan sulfate alone suggests MPS III, keratan sulfate alone suggests MPS IV, and dermatan sulfate alone suggests MPS VI. However, as urinary GAG assays are not diagnostic of a specific MPS, enzyme activity assays must be performed to confirm the diagnosis.

It should be emphasized that a negative urinary GAG test, even using a quantitative assay, does not necessarily rule out the diagnosis of an MPS disorder. False-negative results may occur due to dilute sample, variations in GAG excretion over time, and overlap in ranges between affected and unaffected patients. Mahalingam et al. quantitated urinary GAG levels in 91 healthy children and 219 children with MPS [21]. The mean urinary GAG level was 2.5 ± 2.09 mg/mmol creatinine (range, 0.45–9.96) in the control group and 12.64 ± 12.83 mg/mmol creatinine (range, 0.5–78.4) in the MPS group. Therefore, if clinical suspicion of MPS is high but the urinary GAG level is normal, additional diagnostic testing should be pursued (Fig. 4). Note also that urinary GAG testing cannot be used to determine the MPS II carrier status of a female because the levels observed in carriers are typically within the range observed in non-carrier females [39].

### Enzyme activity

When urinary GAG levels are elevated, or if there is a strong clinical suspicion of MPS II, enzyme activity testing should be conducted. Although enzyme activity can be measured in cultured fibroblasts, the standard samples are leukocytes, plasma, or serum [44]. Enzyme assays based on the analysis of dried blood spots are also being performed and are useful in areas where it is difficult to collect and/or transport liquid samples [6, 9]. Absent or very low I2S activity is diagnostic of MPS II. A second sulfatase should also be measured in the patient's sample to rule out multiple sulfatase deficiency, an LSD that affects the entire sulfatase family [10].

Although enzyme assays are considered the gold standard for diagnosis of MPS, they have several limitations. First, there is no correlation between measurable I2S activity, as determined by routine diagnostic assays, and eventual phenotype [33]. Second, enzyme activity is not reliable for the identification of female carriers because of an overlap in activity ranges between carrier and non-carrier females [18, 39].

### Genetic testing

Genetic testing of the *IDS* locus is the only reliable way to identify female carriers of the disease, a critical factor in family planning decisions [18]. To date, over 330 alterations in the *IDS* gene have been reported in MPS II patients, many of which are seen only within a single family [40]. Unfortunately, the genetic heterogeneity associated with MPS II and the private nature of many mutations present major obstacles to genotype–phenotype correlation. Although uncommon, the MPS II phenotype can vary even among family members who share the same *IDS* mutation [12]. It is known that deletions or rearrangements of the *IDS* gene that completely abolish I2S transcript production will result in the severe phenotype. This information is valuable to provide to families when it is available. The phenotypic effects of other types of mutations are much more difficult to predict.

### Preimplantation genetic diagnosis and prenatal testing

Preimplantation genetic diagnosis for MPS II has been reported in three Israeli families with successful outcomes in all three, but this technique is not yet widely available [1]. Prenatal diagnosis via I2S enzyme assays or molecular testing in chorionic villi or amniotic fluid cells is available at some centers and can be offered to all couples with a family history of MPS II to allow for informed decision making about pregnancy termination [17]. Sex determination of the fetus is not adequate for a prenatal diagnosis, given that a male offspring of a carrier mother has a 50% chance of being unaffected.

Beyond family planning decisions, prenatal diagnosis has also allowed for early initiation of ERT in other forms of MPS, and sibling pair case-study data suggest that the benefit of ERT was clearly greater in the sibling treated from infancy than in the sibling treated as a young child [25]. Similar conclusions about benefits of early treatment have been reached for MPS II and early treatment has been recommended in the recent European guidelines [37]; however, this has not been evaluated in clinical trials.

### Choosing a laboratory for diagnostic testing

Clinicians should be aware that few laboratories perform the diagnostic assays described above, and that values can vary from laboratory to laboratory. The latter is particularly true for urinary GAG testing [20]. Genetic testing and prenatal testing for MPS II are not commonly performed, so it is important to ensure that the laboratory used is accredited and experienced in these analyses. Listings such as GeneTests [27] may be helpful in this regard.

### Looking forward: newborn screening

The eventual goal for the early treatment of MPS II is newborn screening; progress on this front has been reviewed by Marsden and Levy [23]. Chamoles et al. [4] first demonstrated that many lysosomal enzymes remain active in dried blood spots and can be assayed after rehydration in a suitable reaction buffer. Since that time, assays for lysosomal enzyme activity, including I2S activity, in dried blood spots that may be suitable for newborn screening have been developed [13]. Pilot population-based newborn screening using any of these methods has not yet been reported. Until newborn screening for MPS II becomes widespread, it remains in the hands of aware and astute pediatricians, family practice physicians, and pediatric specialists to recognize and refer patients with suspected MPS II as early as possible.

## After the diagnosis: multidisciplinary management of MPS II

When a diagnosis of MPS II is confirmed or is strongly suspected, prompt referral to a clinical geneticist is a necessity in order to ensure timely treatment. Nonetheless, the pediatrician or family practice physician has a key role to play in the multidisciplinary management of the child with MPS II (reviewed by Muenzer et al. [28]). Patients and caregivers are often overwhelmed with the number of pediatric subspecialties that are involved in care, including anesthesiology, cardiology, neurodevelopment, neurosurgery, ophthalmology, orthopedics, otorhinolaryngology, and pulmonology. Supportive services such as physiotherapy, occupational therapy, speech therapy, audiology, dentistry, and behavioral therapy are also involved. The pediatrician or family practice physician can help facilitate care among the many specialists the family must interact with and can provide a consistent medical home for the patient.

It is important to note that because of the airway obstruction, pulmonary involvement, and spinal cord compression associated with MPS II, any surgical or screening procedures that require general anesthesia or sedation should be performed only in a medical center that has extensive experience in handling children with MPS II [31].

## Conclusion

Mucopolysaccharidosis II is a rare genetic disorder that affects multiple organ systems and reduces life expectancy. Early diagnosis and referral are critical to help optimize patient outcomes. ERT with recombinant human I2S enzyme is available, and early treatment may result in greater clinical benefit, although more long-term data are needed to fully assess the benefits of early treatment. A clinical trial in patients under 5 years of age is currently ongoing. Clinical suspicion of MPS II should be raised when a child presents with a cluster of characteristic signs and symptoms or a history of early and frequent surgeries. Not all patients with MPS II will have all signs and symptoms, and severity can vary widely. Urinary GAG testing is useful for screening, although a negative result does not rule out MPS II. Diagnosis is confirmed by the measurement of I2S activity, usually in blood. Genetic testing is the only reliable method for carrier identification. If the standard diagnostic assays are all within normal range but the child displays MPS-like signs and symptoms, referral to a clinical geneticist is strongly encouraged, as other genetic syndromes share features with MPS II. Once a diagnosis is made, multidisciplinary management is critical and should involve the pediatrician or family practice physician.

## Abbreviations

ERT: Enzyme replacement therapy
GAG: Glycosaminoglycan
I2S: Iduronate-2-sulfatase
LSD: Lysosomal storage disease
MPS: Mucopolysaccharidosis
